# South West Surgeons

**Published:** 1963-10

**Authors:** 


### SOUTH WEST SURGEONS

The Spring Meeting of the Surgical Club of South West England was held in Torquay. Mr. H. J. Fuller of Bath presided over this first visit of the Club to Torbay Hospital on the 26th and 27th April, 1963. During the whole Meeting there was an excellent static display of specimens, instruments and X-rays. Papers were read by Mr. H. P. Guerrier, Mr. A. M. Gardiner and Mr. P. J. Monks. On Friday morning and in the afternoon 40 patients were shown. With such a wealth of material there was little time for discussion, but the individual members were able to talk about the cases as they saw them.

On Saturday morning Mr. A. P. Holden demonstrated skin grafts and Mr. D. H. Moore of Exeter gave a paper on "Hospital Sepsis in the Torquay area". During the Meeting all were impressed with the very clear photographs and slides. They were taken with a camera developed by Mr. A. M. Gardiner. This unit is now on the market. It is easy to use and gives most excellent results in the hands of the unskilled.

